# On the Statistical Size Effect of Cast Aluminium

**DOI:** 10.3390/ma12101578

**Published:** 2019-05-14

**Authors:** Roman Aigner, Sebastian Pomberger, Martin Leitner, Michael Stoschka

**Affiliations:** Christian Doppler Laboratory for Process based Component Design, 8700 Leoben, Austria; sebastian.pomberger@unileoben.ac.at (S.P.); martin.leitner@unileoben.ac.at (M.L.); michael.stoschka@unileoben.ac.at (M.S.)

**Keywords:** cast aluminium, fatigue assessment, shrinkage pores, statistical distribution, extreme value statistics, highly-stressed volume

## Abstract

Manufacturing process based imperfections can reduce the theoretical fatigue strength since they can be considered as pre-existent microcracks. The statistical distribution of fatigue fracture initiating defect sizes also varies with the highly-stressed volume, since the probability of a larger highly-stressed volume to inherit a potentially critical defect is elevated. This fact is widely known by the scientific community as the statistical size effect. The assessment of this effect within this paper is based on the statistical distribution of defect sizes in a reference volume $V_{0}$ compared to an arbitrary enlarged volume $V_{\alpha }$. By implementation of the crack resistance curve in the Kitagawa–Takahashi diagram, a fatigue assessment model, based on the volume-dependent probability of occurrence of inhomogeneities, is set up, leading to a multidimensional fatigue assessment map. It is shown that state-of-the-art methodologies for the evaluation of the statistical size effect can lead to noticeable over-sizing in fatigue design of approximately 10%. On the other hand, the presented approach, which links the statistically based distribution of defect sizes in an arbitrary highly-stressed volume to a crack-resistant dependent Kitagawa–Takahashi diagram leads to a more accurate fatigue design with a maximal conservative deviation of 5% to the experimental validation data. Therefore, the introduced fatigue assessment map improves fatigue design considering the statistical size effect of lightweight aluminium cast alloys.

## 1. Introduction

In order to ensure conservative fatigue design of heterogeneous material, size effects have to be assessed properly, since they significantly influence the fatigue strength of engineering components. A study in [1] separates the size effects into statistical [2,3,4], geometrical [1,5,6], technological [7,8] and surface technology [9] contributions. The statistical size effect, whose improved assessment is the aim of this work, leads to a decrease of fatigue strength with elevated size of the structure or specimen due to increased probabilities of critical defect sizes. Griffith’s investigations in [10] revealed such an effect, due to the presence of microcracks. One of the most used statistical models is the weakest link concept of Weibull [11]. Accordingly, the probability of a structure or specimen to inherit a critical defect size elevates in line with the volume of the component—see [12]. This concept was later confirmed to be valid for the high cycle fatigue fracture by Heckel in [13]. A more recent study [14] revealed similar effects in the very high cycle fatigue (VHCF) region, pointing out the need for using large specimens in VHCF testing, in order to consider critical defect sizes. Another method to characterize the statistical size effect was proposed by Carpinteri in [15,16,17]. Hereby, a fractal geometry approach is utilized for the characterization of the fracture surfaces, assuming them to possess non-integer dimensions. By modelling the self-similar fracture surfaces, mechanical properties depending on the fractal dimension can be deduced—see [15,16,17]. Hence, the Basquin equation [18] is modified invoking the fractal approach. Another methodology to assess the statistical size effect is given in [19]. Hereby, the highly-stressed volume, defined as the volume which is stressed by over 95% of the peak stress value, is considered in an exponential approach—see Equation (1):

(1) $$\sigma_{\mathrm{LLF}}=B\cdot V_{95}^{-a},$$

with *B* and *a* being material dependent coefficients and $V_{95}$ the 95% highly-stressed volume, with respect to the maximum stress. In order to evaluate the highly-stressed volume, a numerical procedure to evaluate stress integrals is provided in [20]. Sonsino [21] proposed a similar volumetric model, invoking the 90% highly-stressed volume, $V_{90}$ and the Weibull exponent κ—see Equation (2):

(2) $$\frac{\sigma_{\mathrm{LLF},1}}{\sigma_{\mathrm{LLF},2}}={\left(\frac{V_{90,2}}{V_{90,1}}\right)}^{\frac{1}{\kappa }}.$$

The Weibull exponent is a material dependent coefficient, which characterizes the slope in the double logarithmic $\sigma_{a,V_{i}}-V_{i}$ plot and therefore the decrease in fatigue strength at an increased highly-stressed volume. Its value is listed for various material classes in a common fatigue design guideline—see [22]. In case of cast aluminium, κ is proposed to possess a value of ten [1,22]. It has to be mentioned that, with increasing highly-stressed volume, a saturation effect was observed, such that no noticeable further decrease in fatigue strength can be observed at volumes $V\geq V_{\kappa \infty }$. The threshold volume $V_{\kappa \infty }$ is proposed to be $8000\;{\mathrm{mm}}^{3}$ for cast material in [23]. Investigations in [24] modified this approach, invoking the highly-stressed surface instead of an highly-stressed volume, since surface near defects are revealed to be more critical in terms of fatigue life, as also stated in [25,26,27,28].

The validation of the different presented models for the size effect, respectively the corresponding fatigue strength assessment, reveals that the weakest link model and the volumetric model match the experimental fatigue data best—see [29]. Furthermore, it is stated within the same study that both the weakest link model as well as the volumetric model lead to similar results. Preliminary studies in [30,31], which investigate the statistical size effect of an aluminium alloy containing artificial surface defects, reveal the volume approach to be more suitable than the surface approach. Thus, the investigation of the statistical size effect in this study is conducted by means of the volumetric model proposed by Sonsino as given in [23]. Kitagawa and Takahashi proposed in [32] a fatigue assessment method for material inheriting small cracks respectively heterogeneities. Due to its sound applicability, the methodology proposed by Kitagawa and Takahashi has been utilized for the fatigue strength assessment of imperfect materials in several preliminary studies [33,34,35,36,37].

Hereby, the long-life fatigue strength can be assessed invoking two major boundaries. On the one hand, there is the fatigue strength of the defect free material $\Delta \sigma_{0}$ with crack sizes tending towards zero and on the other hand, when defect respectively crack sizes *a* become sufficiently large, there is the long crack threshold $\Delta K_{th,lc}$. The long life fatigue strength $\sigma_{a}$ of a material inheriting flaws can therefore be estimated fracture mechanically assessed by Equation (3):

(3) $$\Delta \sigma_{\mathrm{LLF}}=\frac{\Delta K_{th,lc}}{Y\sqrt{\pi a}},$$

with *Y* being the geometry factor, taking the defect size, shape and location into account—see [38]. A qualitative study on the influence of the defect orientation and location on the stress concentration factor is given in [39]. The original Kitagawa–Takahashi diagram KTD was later modified by El Haddad in [40,41], such that the fatigue strength steadily decreases with elevating defect sizes *a*.

**Figure 1:**
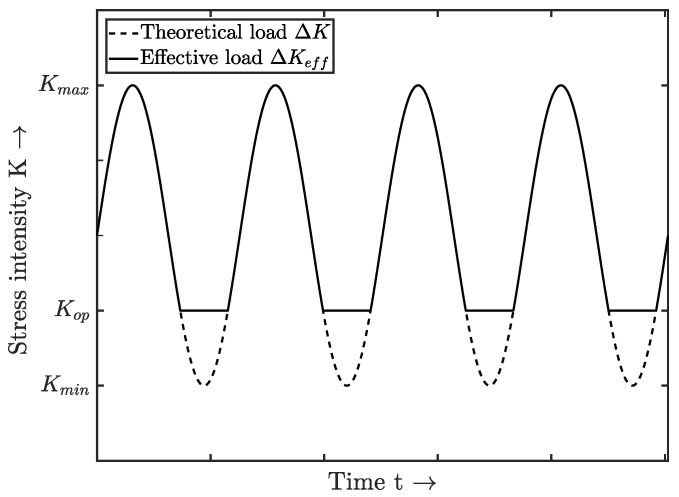
Effective load stress intensity factor due to crack closure effects.

A further modification of the KTD is given by the implementation of the crack resistance curve (R-Curve), as introduced by Chapetti in [42]. The cyclic R-curve characterizes the resistance of the material to crack initiation, respectively propagation. Therefore, the crack resistance rises from the intrinsic threshold $\Delta K_{th,eff}$ to the long crack threshold $\Delta K_{th,lc}$ with elevating crack length Δa due to crack closure effects [43]. Crack closure effects lead to premature physical contact of the crack shoulders, such that the original load range $\Delta K=K_{max}-K_{min}$ is reduced to an effective one $\Delta K_{eff}=K_{max}-K_{op}$—see Figure 1.

The most distinguished crack closure effects are the roughness- [44,45], plasticity- [46,47], and oxide-induced [48,49] effects. Hence, the composition of aforementioned contributions leads to a crack resistance curve. The R-curve can be characterized by an exponential law as proposed in [50]. A more elaborated procedure to describe the course of the R-curve was proposed by Maierhofer et al. in [51]. This methodology takes the contributions $\nu_{i}$ of the very crack closure effects into account. Each effect with index *i* is assumed to be completely built up after reaching the crack extension length $l_{i}$—see Equation (4):

(4) $$\Delta K_{th,\Delta a}=\Delta K_{th,eff}+\left(\Delta K_{th,lc}-\Delta K_{th,eff}\right)\left[1-\sum_{i=0}^{n}\nu_{i}\cdot \exp\left(-\frac{\Delta a}{l_{i}}\right)\right],$$

with

$$\sum_{i=0}^{n}\nu_{i}\equiv 1.$$

Since the contributions of the very crack closure effects cannot be defined a priori due to influences of the specimen geometry [52], this model represents a potent tool to qualitatively assess the elevating crack resistance with increasing crack length. Thus, the cyclic R-curve can be implemented in the KTD by inserting Equation (4) in the Kitagawa–Takahashi relationship—see Equation (3). A preliminary study in [53] reveals this approach to be suitable to assess the mean stress sensitivity of both imperfect and near defect free material. Hereby, a three-dimensional KTD is set up by implementing a mean stress sensitivity approach, provided in a common guideline [22] and a generalized R-curve considering varying load stress ratios, as proposed in [54].

Hence, the extension of the Kitagawa–Takahashi diagram by the crack resistance R-curve is suitable to assess both physically short and long cracks in terms of fatigue strength, where the crack length *a* is substituted by an equivalent flaw size $\sqrt{area}$ if sharp defects such as micro shrinkage pores are considered. The $\sqrt{area}$ parameter was introduced by Murakami in [55] and is given by the square root of the projected area of the fatigue fracture initiating heterogeneity, measured perpendicular to the maximum principal load direction. Since a preliminary study in [56] revealed that the $\sqrt{area}$ parameter correlates well with the stress field around the inhomogeneity, this parameter is further utilized in this work for the estimation of defect sizes. The defect size measurement methodology invoked in this work is in detail described in [57,58,59]. According to investigations in [60,61], the statistical distribution of defect sizes can be characterized by the limiting extreme value distribution of the Lognormal (LN) distribution, namely the Gumbel or Extreme Value type one distribution (EV) [62,63]. Since the Generalized Extreme Value (GEV) distribution [64] inherits the Extreme Value distributions of type one, two and three (Gumbel, Frechet and Weibull), the GEV distribution is also applicable for the characterization of fatigue fracture initiating defect sizes—see [65]. The cumulative distribution function of the GEV is given in Equation (5):

(5) $$\begin{array}{c} P\left(\sqrt{area}\right)=\exp\left\{-{\left[1+\xi \left(\frac{\sqrt{area}-\mu }{\delta }\right)\right]}^{-\frac{1}{\xi }}\right\} \end{array}$$

and

(6) $$\begin{array}{c} P_{Occ}=1-P\left(\sqrt{area}\right). \end{array}$$

Its course is defined by the location μ, scale δ and shape ξ parameter which are evaluated by means of a maximum likelihood method, as proposed in [66,67]. Another methodology for calculating the maximum inclusion, inherited in enlarged geometries, is given in [68,69]. By evaluating the most extremal defect in a given reference volume $V_{0}$, the obtained defect sizes are to be Gumbel distributed, with the cumulative distribution function P($\sqrt{area}$) of Equation (7), characterized by its location- μ and scale parameter δ:

(7) $$P\left(\sqrt{area}\right)=exp\left\{-exp\left[-\frac{\sqrt{area}-\mu }{\delta }\right]\right\}.$$

This methodology, also referred to as extreme value inclusion rating *EVIR*, enables the prediction of the size of a potentially critical inclusion with a spatial extent $\sqrt{area}\left(T\right)$ in an enlarged control Volume $V_{\alpha }$, which is defined by the return period *T*—see Equation (8):

(8) $$\begin{array}{c} \sqrt{area}\left(T\right)=\mu -\delta \cdot ln\left[-ln\left(1-\frac{1}{T}\right)\right], \end{array}$$

with

(9) $$\begin{array}{c} T=\frac{V_{\alpha }}{V_{0}}. \end{array}$$

Hereby, the very fatigue fracture initiating defect sizes of every specimen are sorted and indexed with j=1,⋯,n. Subsequently, the cumulative probability $F_{j}$ can be derived for each sample *j* invoking Equation (10). Accordingly, a reduced variate $y_{j}$ can be estimated, as depicted in Equation (11):

(10) $$\begin{array}{c} F_{j}=\frac{j}{n+1} \end{array}$$

and

(11) $$\begin{array}{c} y_{j}=-ln\left[-ln\left(F_{j}\right)\right]. \end{array}$$

Furthermore, Murakami [68] proposes an empirical formula for the defect based fatigue strength of various material types—see Equation (12). Since the fatigue strength of metallic materials is revealed to correlate with the Vickers hardness HV, Murakami invokes this material constant, in line with two fit parameters, $C_{1}$ and $C_{2}$. Hereby, $C_{1}$ acts as a geometry factor, depending on the location of the defect, being 1.43 for a surface-near and 1.56 in case of a subsurface defect. The coefficient $C_{2}$ is regularly a priori defined to possess a value of 120. Nevertheless, preliminary studies [57,70] propose a factor $C_{2}$ of zero for similar materials, induced by the deviation in defect size measurement methodologies:

(12) $$\sigma_{\mathrm{LLF}}=C_{1}\frac{C_{2}+\mathrm{HV}}{{\sqrt{area}}^{\frac{1}{6}}}.$$

Although the statistical size effect due to natural defects is a well researched field, the probabilistic fatigue assessment method presented in this work is a potent tool for conservative fatigue design. Hence, the focus of this work lies on the evaluation of the statistical distribution of fatigue fracture initiating defect sizes in an arbitrary highly-stressed volume and its impact on the fatigue strength. Finally, the presented fatigue assessment map facilitates fatigue design of parts inheriting manufacturing process based imperfections with improved accuracy in terms of probabilistic assessment.

## 2. Investigated Material

The specimens are taken out from two positions, *A* and *B* from an engineering component made of EN AC-46200 in T6 heat treatment. The nominal chemical composition in line with the standard given in [71] of this aluminium alloy is listed in Table 1. In order to build up a KTD, the fatigue strength of the defect free material has to be evaluated. Therefore, additional specimens are taken out from the same positions with additional preliminary hot isostatic pressing (HIP) treatment.

**Figure 2:**
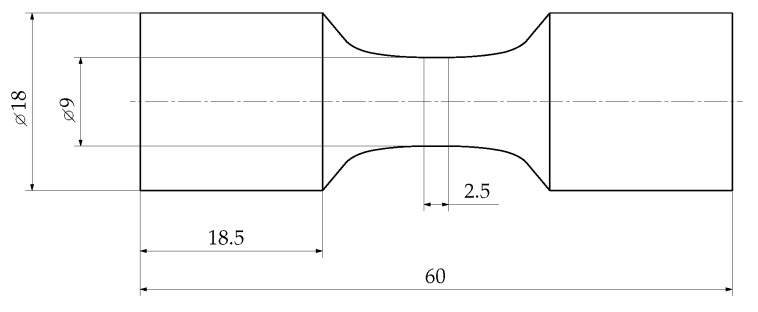
HCF specimen geometry A.

**Figure 3:**
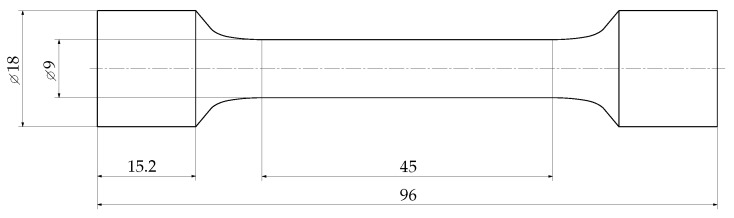
HCF specimen geometry B.

The microstructural properties in terms of the dendrite arm spacing (DAS) are evaluated by a procedure proposed in [72], since the DAS correlates well with both quasi-static [73,74,75] and fatigue properties [76,77] due to its dependency with inter-dendritic shrinkage pore sizes [57]. The investigations of the DAS reveal a minor deviation of just five percent between sampling position A and B, such that technological size effects are negligible at these two positions. Thus, differently sized specimens can be taken from these two positions to evaluate the statistical size effects. Furthermore, the specimens have been designed by numerical shape optimization of the fillet region, such that a minor stress concentration factor of just 1.02 is not exceeded. This enables a variation of the highly-stressed volume by scaling the length $l_{const}$ of the constant specimen diameter, where the general shape of the specimen is kept similar. The high cycle fatigue (HCF) specimen geometry type A and B are displayed in Figure 2 and Figure 3, respectively. As depicted in Figure 3, the length of the constant testing diameter $l_{const}$ of specimen B is significantly enhanced by a factor of 18 with respect to specimen geometry A, to force a significant statistical size effect. Subsequently, the two specimen types are only labelled in relation to their different geometry as A, small-sized reference sample, and B, enhanced highly-stressed volume size.

**Figure 4:**
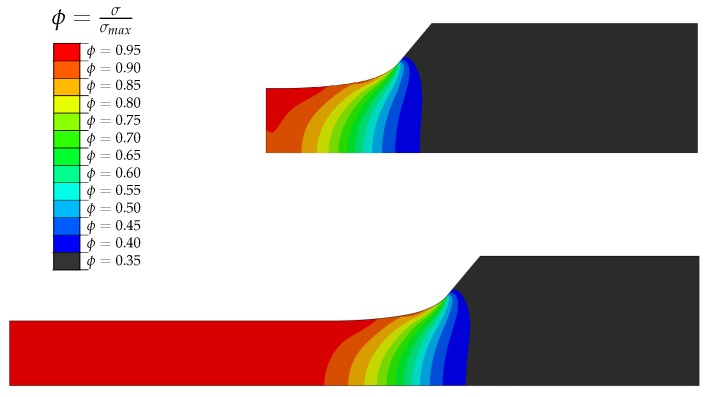
FEM analysis of specimen types A and B.

The highly-stressed volume is furthermore numerically evaluated. In line with the subsequent testing procedure, the linear-elastic finite element analysis is conducted with an unified uni-axial tension load. Hereby, the numerical model is set up invoking eight node axisymmetric CAX8R elements. In Figure 4, the results of the numerical analysis are depicted, showing the normalized maximum principle stress.

The evaluation of the 95% highly-stressed volumes hence leads to a ratio between geometry B and A of $V_{95,B}/V_{95,A}\approx 10$, as described in [57]. Hence, the highly-stressed volume of position A is $V_{95,A}$ considered as reference volume $V_{0}$, where the increased volume of position B is in the following referred to as highly-stressed volume $V_{1}$.

## 3. Results

### 3.1. Fatigue Strength

The fatigue strength of both specimens is evaluated at the same resonant testing machine, utilizing a testing frequency of just beneath 120 Hz and fully reversed/tension compression loading. The run out number was preliminarily defined to be ten million cycles in order to ensure a statistically assessed long life fatigue strength. As proposed in prior studies [78] and applied in [4,70], the slope of the S/N-curve in the long life region $k_{2}$ is considered to scale with the slope in the finite life $k_{1}$, such that $k_{2}=5\cdot k_{1}$—see [78]. The evaluation of failures in the finite life region is conducted by the statistical procedure given in the standard [79]. The long life fatigue strength is assessed by means of the arcsin$\sqrt{p}$ methodology—see [80].

**Figure 5:**
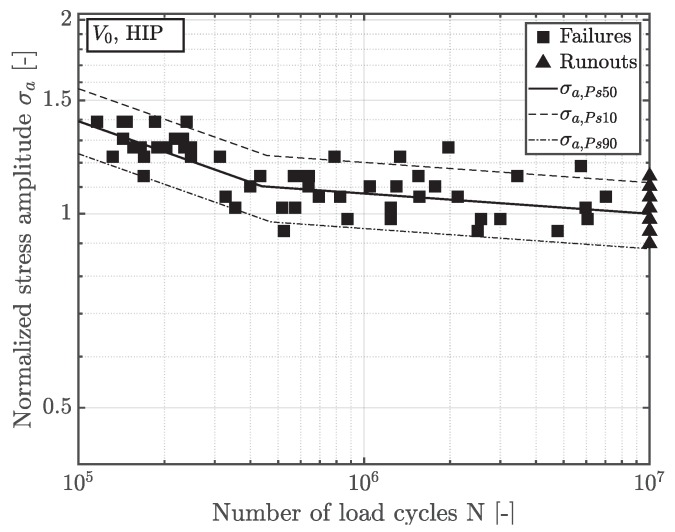
S/N curve of near defect free position A.

In order to evaluate the fatigue strength of the defect free material, specimens of geometry A are taken from an HIP treated component. Again, it was ensured by DAS-evaluation that no technical size effect occurred at this position. The fatigue data as well as the statistically estimated slopes in the finite and long life region as well as the scatter bands are depicted in Figure 5. The fatigue strength of the near defect free HIP material at ten million load cycles is considered as a reference value in the further work.

**Figure 6:**
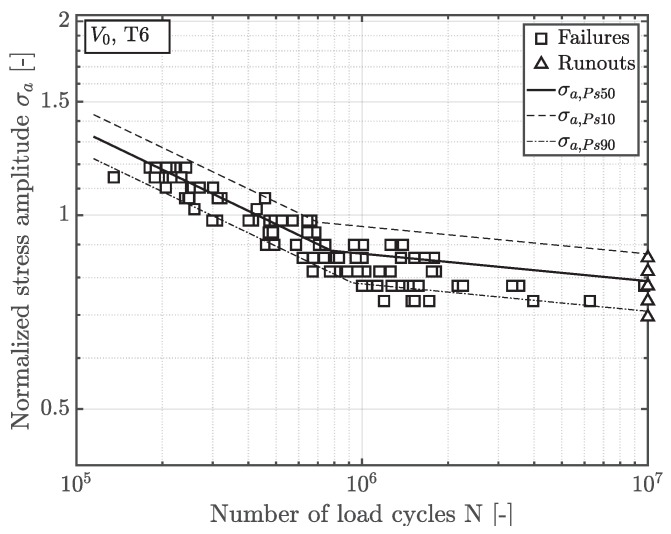
S/N curve of position A.

**Figure 7:**
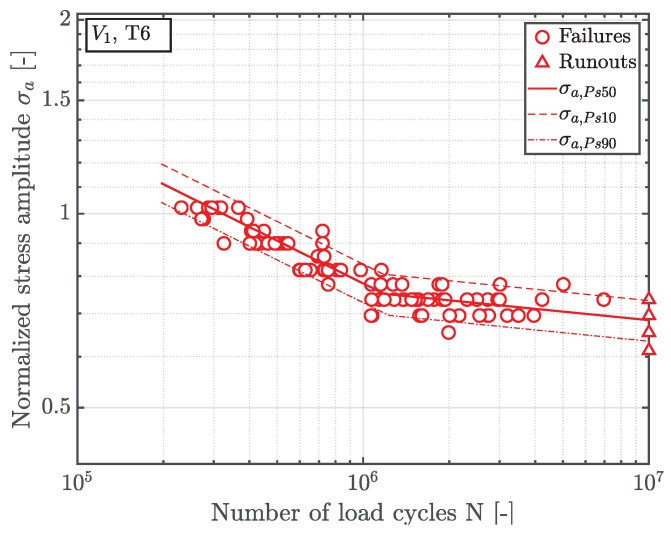
S/N curve of position B.

The statistically evaluated S/N-curve of position A, inheriting a highly-stressed volume $V_{0}$, is depicted in Figure 6, along with the 90 and 10 percent scatter band. All fatigue data is normalized by the fatigue strength of the near defect free material of position A. Thus, the long life fatigue strength $\sigma_{\mathrm{LLF}}$ decrease by about 20% in the presence of shrinkage pores. Since the highly-stressed volume $V_{1}$ of position B is majorly enhanced, its fatigue strength reveals a significant statistical size effect in terms of an additional decrease in fatigue strength by approximately fifteen percent—see Figure 7.

The evaluated slopes $k_{1}$ of the imperfective specimens in the finite life correlate well with a value of about 4.5. Thus, the derived slopes $k_{2}$ in the long life region match with the proposed value of 22 in the standard [81]. It has to be mentioned that the scatter band of the specimens of position B is revealed to be minor with respect to the specimens of position A, where the number of cycles $N_{T}$ of the transition knee-point is somewhat enhanced in position B—see Table 2.

The long life fatigue strength $\sigma_{\mathrm{LLF}}$ is additionally listed in Table 2, where all fatigue data are normalized by the fatigue strength of near defect free position A at ten million load cycles, considering a probability of survival $P_{S}$ of 50%. Additionally, the slope of the finite life region $k_{1}$, the number of load cycles for the transition point $N_{T}$, and the statistically evaluated fatigue scatter band $T_{S}$ is given in Table 2.

**Figure 8:**
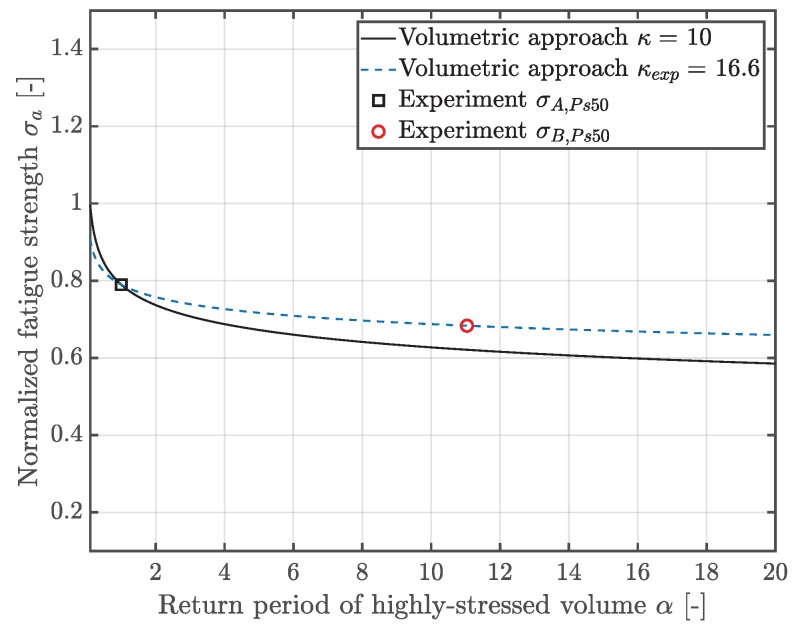
Volumetric model with the theoretical and experimentally deduced Weibull factor κ.

Thus, the statistical size effect can be assessed by means of the volumetric model, revealing a deviation of 8% with respect to the experimentally observed fatigue strength. Furthermore, a local experimental Weibull factor $\kappa_{exp}$ can be derived, revealing a significantly enhanced value of 16.6, rather than the originally proposed value of 10. The volumetric model, along with the experimental fatigue test results at 50% probability of survival $P_{S}$, is depicted in Figure 8. This significant deviation points out the need for more precise methodologies for the fatigue assessment of defective materials.

### 3.2. Fractography

In addition, the fatigue fracture initiating defect sizes of the HCF-specimens are investigated subsequent to the testing by means of digital optical microscopy respectively scanning electron microscopy.

In contrast to the method proposed by Murakami in [68], where “effective” heterogeneity sizes are measured, the defect sizes are evaluated rather form fitting in this work. Thus, the investigation of the spatial extent of a flaw will lead to comparably smaller sizes. Nevertheless, this methodology is of utmost importance in order to minimize any distortion regarding the subsequent statistical analysis.

**Figure 9:**
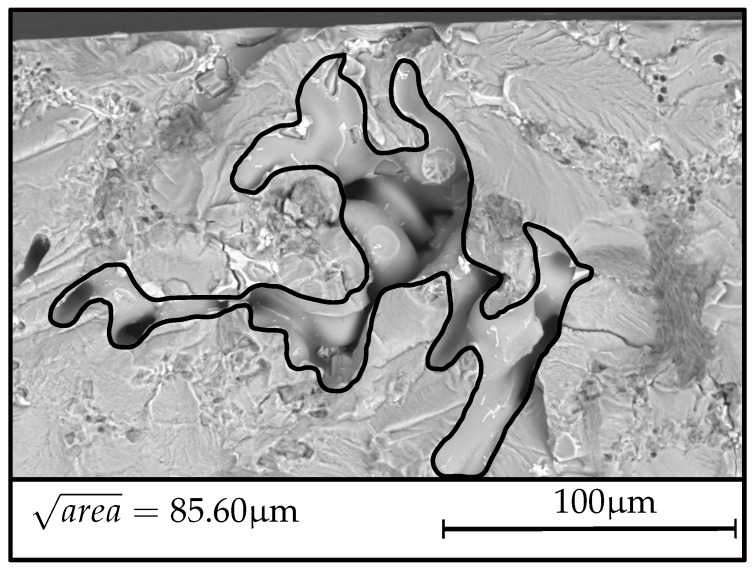
Representative fracture initiating defect at specimen geometry A.

**Figure 10:**
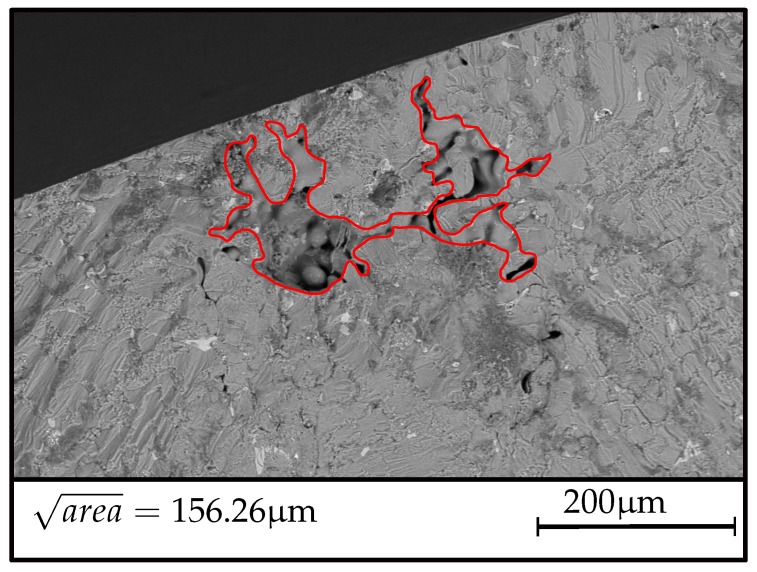
Representative fracture initiating defect at specimen geometry B.

Figure 9 displays the fatigue fracture surface of a representative specimen of position *A*, revealing an equivalent crack size $\sqrt{area}$ of just over 85 μm. Accordingly, Figure 10 depicts a fracture surface and the critical defect as the very point of crack initiation at geometry *B*. The evaluated critical defect size displayed in Figure 10 is significantly enhanced with respect to average inhomogeneity sizes in position A, in line with the general presumption of the statistical size effect. Hence, the evaluated decrease in terms of fatigue strength of position B is caused by the elevated spatial extent of pre-existent microcracks respectively micro shrinkage pores.

In order to statistically evaluate the most critical defect sizes, the generalized extreme value distribution with the cumulative distribution function (CDF) given in Equation (5) is invoked, as proposed in [65]. Additionally, a Kolmogorov–Smirnov test for the goodness of fit is utilized for the statistical assessment of the distributions—see [82]. Considering a reference volume $V_{0}$, the course of the probability of occurrence $P_{Occ}$ of a heterogeneity with a critical defect is characterized by the location, shape and scale parameters of the reference distribution *P*. Furthermore, the cumulative distribution function of a α-times enlarged volume $V_{\alpha }$ can be derived as the following [62]:

(13) $$\begin{array}{c} V_{0}∼P\left(\sqrt{area};\mu ,\delta ,\xi \right)=\exp\left\{-{\left[1+\xi \left(\frac{\sqrt{area}-\mu }{\delta }\right)\right]}^{-\frac{1}{\xi }}\right\}, \end{array}$$

(14) $$\begin{array}{c} V_{\alpha }∼P^{\alpha }, \end{array}$$

which leads to

(15) $$\begin{array}{c} P^{\alpha }=\exp\left\{-{\left[1+\xi \left(\frac{\sqrt{area}-\left(\mu +\frac{\delta }{\xi }\left(\alpha^{\xi }-1\right)\right)}{\delta \alpha^{\xi }}\right)\right]}^{-\frac{1}{\xi }}\right\}, \end{array}$$

(16) $$\begin{array}{c} V_{\alpha }∼P\left(\sqrt{area};\mu +\frac{\delta }{\xi }\left(\alpha^{\xi }-1\right),\delta \alpha^{\xi },\xi \right). \end{array}$$

**Figure 11:**
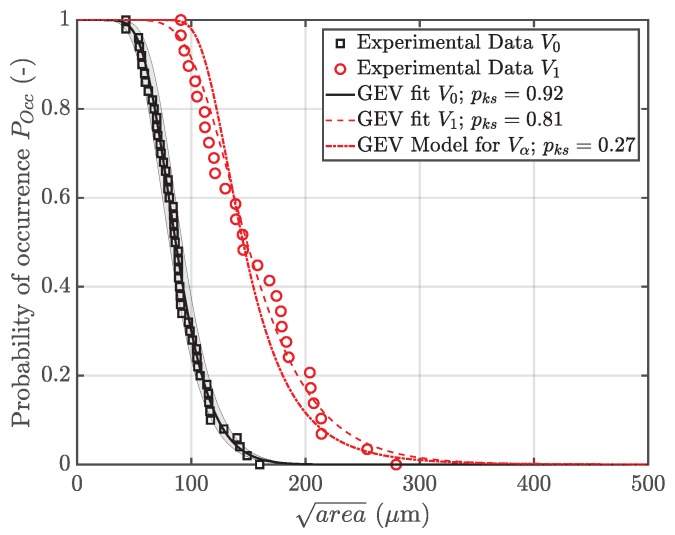
Probability of occurrence of critical defect sizes in specimen geometry A and B.

Thus, the parameters for the critical defect size distribution in an α-times greater volume $V_{\alpha }$ can be easily derived, based on the distribution of the reference volume $V_{0}$—see Equation (13). Figure 11 depicts the probability of occurrence $P_{Occ}$ of a flaw with a given size in both specimen geometries *A* and *B*. Furthermore, the parameters of each distribution are statistically estimated by means of a maximum likelihood function, as proposed in [66].

Finally, the derived model for the generalized extreme value distribution in a volume $V_{\alpha }$ is displayed in Figure 11. As proposed by the numerical investigation of the highly-stressed volumes in each specimen geometry, the factor α is defined to possess a value of ten. As depicted, the critical defect sizes in geometry *B* have minor deviation with respect to the theoretical distribution of $V_{\alpha }$, which is caused by variations of fatigue fracture initiating micropore sizes due to the invoked measurement methodology.

Nevertheless, the critical pore size with a probability of occurrence of 50% matches the experimental data, as listed in Table 3.

### 3.3. Fatigue Assessment

**Figure 12:**
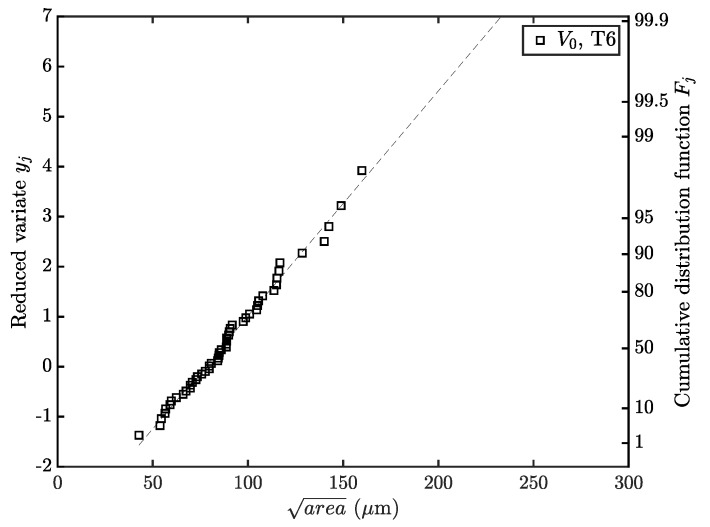
*EVIR* methodology as proposed in [68].

In order to assess the decrease in fatigue strength with elevated probabilities of critical defects, the *EVIR* methodology of Murakami [68] is invoked, as utilized in various preliminary studies [83,84,85]. Hereby, a reduced variate $y_{j}$ is calculated and plotted in a Gumbel probability paper—see Figure 12. Afterwards, the defect in an enhanced volume is computed by invoking the return period T. Thus, the defect sizes in a given control volume and therefore the corresponding fatigue strength are subsequently estimated by the $\sqrt{area}$ approach of Murakami [68,69]—see Table 4.

**Figure 13:**
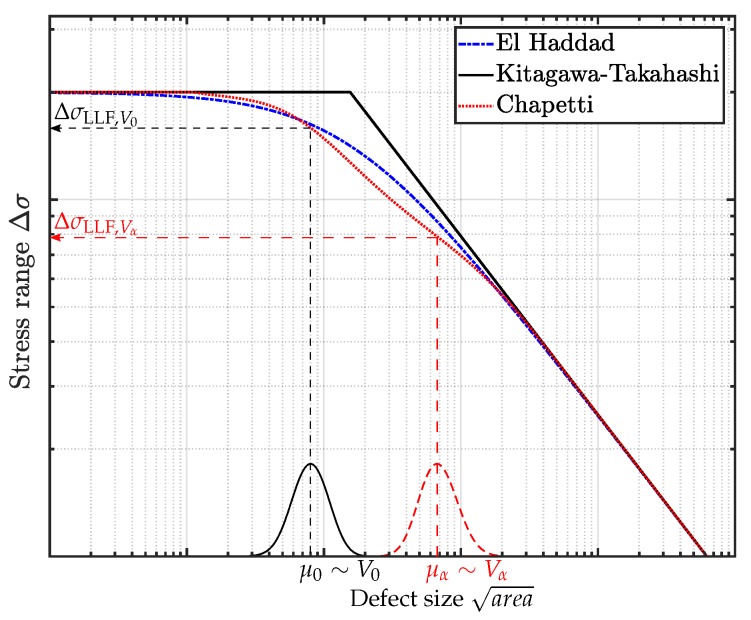
Schematic set up of the fatigue assessment model.

Another possibility to assess the fatigue strength in the presence of microcracks or defects is the Kitagawa–Takahashi diagram. Hence, crack propagation tests were conducted to obtain the materials fracture mechanical threshold value. The single edge notched specimens are taken from identical positions as the HCF specimens in order to minimize microstructural deviations. The testing procedure and extensive specimen preparation, as well as the crack propagation results, are given in detail in a preliminary study—see [37]. As proposed by the author in [37], the crack resistance curve is implemented in the model, such that the KTD is valid both for physically short as well as long cracks. Accordingly, the fatigue strength of specimens with a subsequent HIP treatment characterizes the near defect free material, which defines the left-hand side limit of the KTD, where defect size tends to be zero. Figure 13 displays the schematic set up of the fatigue assessment model. The starting point is given by the defect size in a reference volume $V_{0}$ represented by the size of an inhomogeneity $\mu_{0}$ with a probability of occurrence of $P_{Occ}=50\%$. Subsequently, the fatigue strength is estimated by the R-curve extension of the KTD [42], with respect to the reference volume $V_{0}$. Afterwards, the critical defect size with a similar $P_{Occ}$ in an arbitrary enhanced volume $V_{\alpha }$ is evaluated, invoking Equation (13). The corresponding fatigue strength $\Delta \sigma_{\mathrm{LLF},V_{\alpha }}$ is computed in line with the procedure for the fatigue strength of the reference volume. Hence, a local factor κ depending on the return period α of the highly-stressed volume and the reference defect distribution represented by $\mu_{0}$ can be derived by Equation (17):

(17) $$\kappa \left(\mu_{0},\alpha \right)=\frac{\log(\alpha )}{\log\left(\Delta \sigma_{\mathrm{LLF},V_{0}}\right)-\log\left(\Delta \sigma_{\mathrm{LLF},V_{\alpha }}\right)}.$$

**Figure 14:**
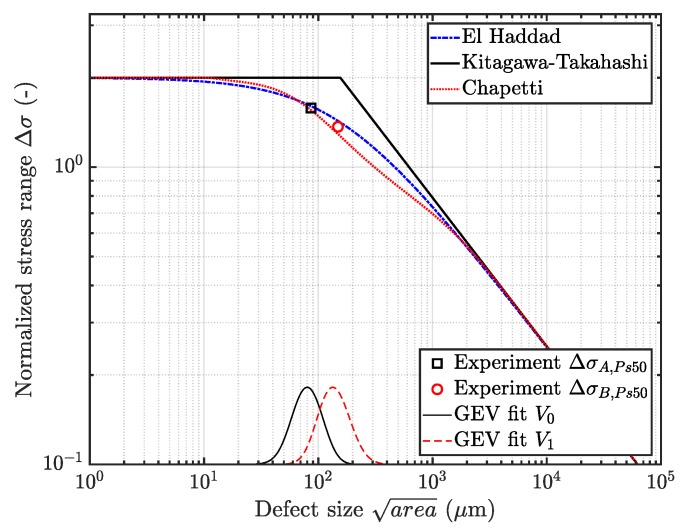
Validation of the fatigue assessment model.

In Figure 14, the set up of the fatigue assessment model based on the KTD is depicted. In line with the S/N-curves, the fatigue range is again normalized by the fatigue strength at ten million load cycles of the near defect free HIP material. Furthermore, Figure 14 displays the experimental results of the fatigue strength at ten million load cycles with a probability of survival of 50%. In addition, the probability density functions of the fatigue fracture initiation shrinkage pore sizes is plotted.

**Figure 15:**
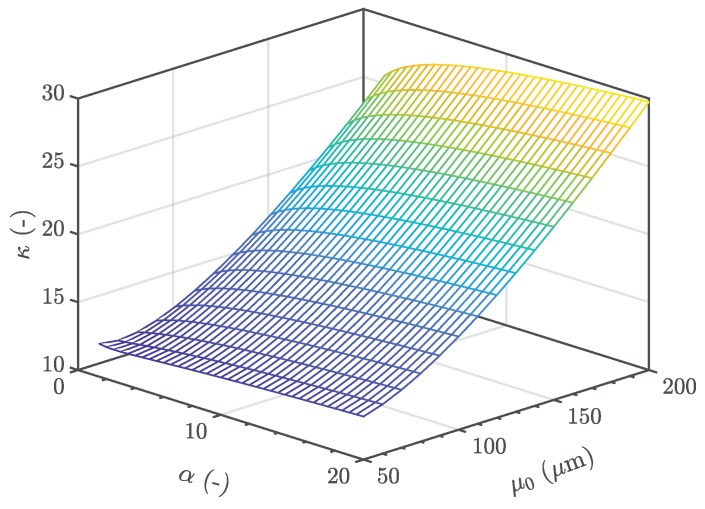
Weibull factor κ depending on the return period α and the local defect population $\mu_{0}$.

In order to estimate the fatigue strength of a given defect distribution, the size of the heterogeneity in an arbitrary control volume $V_{\alpha }$ with a probability of occurrence $P_{Occ}=0.5$ is considered. Subsequently, the corresponding fatigue strength is evaluated, invoking the Chapetti approach as in [42,51] and the model of El-Haddad—see [40,41]. In addition, a local Weibull factor κ can be derived in order to enhance state-of-the-art fatigue design guidelines [22], depending on a reference inhomogeneity population represented by its location parameter $\mu_{0}$, as depicted in Figure 15. Hence, the local κ factor steadily increases with elevated return period α of an highly-stressed volume, which will eventually lead to a saturation of the statistical size effect, in line with conclusions made in preliminary studies—see [23].

Table 4 lists the comparison of commonly invoked fatigue assessment models, accounting for a statistical size effect. Furthermore, the different models are validated by the experimental data of the two specimen geometries with varying highly-stressed volumes. Thus, the volumetric model turns out to be significantly conservative with a decrease in fatigue strength of 8%, leading to over-sizing of critical components in the design process. It has to be mentioned that the volumetric model is based on the statistically estimated fatigue strength of specimen position A, such that position A itself cannot be validated by this approach.

Furthermore, the original $\sqrt{area}$ model of Murakami was modified with a coefficient $C_{2}$ of zero rather than 120, since preliminary studies revealed a proper accordance in fatigue strength assessment with $C_{2}$ equal to zero considering similar cast alloys—see [58,70]. The modified *EVIR* approach of Murakami [68] hence leads to a similar conservative assessment of the fatigue strength as the volumetric approach [21], with 14% for the small-sized and respectively 7% for the large-sized specimen with respect to the experimental results.

**Figure 16:**
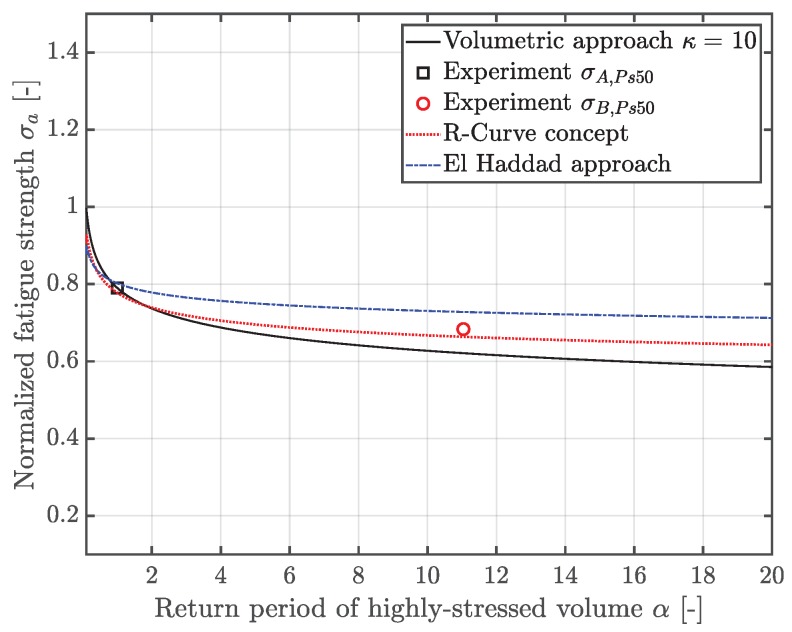
Validation of the fatigue assessment model considering the statistical size effect.

On the other hand, the presented approach in this work, considering the theoretical statistical distribution of flaw sizes in an arbitrary enhanced volume $V_{\alpha }$, reveals a sound correlation with the fatigue data of both geometries A and B with a slightly conservative estimation of the fatigue strength—see Table 4. Finally, a graphical illustration is given in Figure 16. Thus, the fatigue strength of larger components, inheriting an elevated highly-stressed volume, can be properly calculated by the presented model in this work, especially when crack closure effects are considered by means of the implemented R-curve approach.

## 4. Conclusions

This work evaluates the fatigue strength of two differently sized specimens made of EN AC-46200 in T6 condition. Technological size effects are suppressed as the microstructural properties reveal no significant deviation between the investigated sample positions A and B. The presented approach considers both physically short and long crack growth invoking crack closure effects evaluated as the crack resistance R-curve. A probabilistic model of the fatigue fracture initiating flaw sizes in an arbitrary highly-stressed volume is set up, in order to act as input parameter for the subsequent fatigue assessment based on the Kitagawa–Takahashi diagram. Based on the results presented within this paper, the following conclusions can be drawn:

- The return period α possesses a value of approximately ten utilizing the numerically determined highly-stressed volumes of the two specimen geometries A and B.
- Extensive high cycle fatigue tests are statistically evaluated in both the finite as well as the long life region. The fatigue assessment of the empirical HCF data reveals a significant statistical size effect with a decrease in fatigue strength of about 14% in terms of specimen geometry B compared to the reference geometry A.
- The theoretical probability of occurrence of fatigue fracture initiating defect sizes for a given highly-stressed volume is evaluated based on the distribution of critical heterogeneities in a reference volume. The validation of the theoretical distribution with the estimated spatial extents of crack-initiating flaws reveals a minor deviation of just two percent, evaluated for a probability of occurrence of 50%.
- The defect size at a $P_{Occ}=0.5$ subsequently acts as equivalent crack size for the fatigue assessment invoking the Kitagawa–Takahashi diagram with respect to its extensions for short crack growth.
- The validation of the defect based probabilistic fatigue assessment model with the empirical fatigue data of both specimen types with varying highly-stressed volumes reveals that the introduced R-curve concept to assess the fatigue strength best has a conservative deviation of just five percent.

Future work will deal with the implementation of the statistically distributed fatigue strength of the defect free material as well as the long crack threshold, as originally presented in [37], along with a mean stress sensitivity model, as provided in [53], in order to set up a holistic fatigue assessment map.

## Figures and Tables

**Table 1:** Nominal chemical composition of the investigated cast alloy EN AC-46200 in weight percent.

Alloy	Si (%)	Cu (%)	Fe (%)	Mn (%)	Mg (%)	Ti (%)	Al (-)
EN AC-46200	7.5–8.5	2.0–3.5	0.8	0.15–0.65	0.05–0.55	0.25	balance

**Table 2:** Evaluated results of the fatigue tests.

	Position	HT	Volume	$k_{1}$ [-]	$\sigma_{\mathrm{LLF}}$ [-]	$N_{T}$ [-]	$T_{S}$ [-]
	A	HIP	$V_{0}$	7.10	1.00	438.627	1:1.27
	A	T6	$V_{0}$	4.70	0.79	780.732	1:1.23
	B	T6	$V_{1}$	4.54	0.68	11,184.783	1:1.16

**Table 3:** Statistically evaluated and derived distribution parameters for the GEV.

	Position	Volume	μ	δ	ξ	$\sqrt{\mathrm{area}}P_{\mathrm{Occ}=0.5}$ (μm)
	A	$V_{0}$	78.6	21.6	0.11	86
	B	$V_{1}$	134.6	38.6	0.01	148
	B	$V_{\alpha }$	135.0	27.7	0.11	145

**Table 4:** Comparison of the fatigue assessment models.

Position	V	Experiment	Volumetric	Δ	ElHaddad	Δ	Chapetti	Δ	Murakami	Δ
A	0	0.79	Basis	Basis	0.80	+2%	0.78	−2%	0.68 ${}^{a}$	−14%
B	1	0.68	0.63	−8%	0.72	+5%	0.65	−5%	0.64 ${}^{a}$	−7%

${}^{a}$ Modified Murakami approach invoking a coefficient $C_{2}$ of zero.

## Abbreviations

The following abbreviations and symbols are used in this manuscript:

**Abbreviation**	**Description**
VHCF	Very high cycle fatigue
$\sigma_{\mathrm{LLF}}$	Long-life fatigue strength
B,a	Material dependent coefficients of the Kuguel approach
$V_{95}$	95% highly-stressed volume
$V_{90}$	90% highly-stressed volume
κ	Weibull coefficient
$V_{\kappa \infty }$	Threshold volume
$\Delta \sigma_{0}$	Fatigue strength range of the defect free material
$\Delta K_{th,lc}$	Long crack threshold
*Y*	Geometry factor
*a*	Crack length
KTD	Kitagawa–Takahashi diagram
$\Delta K_{th,eff}$	Effective crack threshold
Δa	Crack extension
ΔK	Stress intensity factor range
$K_{max}$	Maximum stress intensity factor
$K_{min}$	Minimum stress intensity factor
$\Delta K_{eff}$	Effective stress intensity factor range
$K_{op}$	Opening stress intensity factor
$\Delta K_{th,\Delta a}$	Crack threshold range in respect of the crack extension
$\nu_{i}$	Weighting factor for crack closure effects
$l_{i}$	Necessary crack elongation for complete build-up of crack closure
$\sqrt{area}$	Defect size parameter
LN	Lognormal distribution
EV	Extreme Value distribution type one
GEV	Generalized Extreme Value distribution
$P(\sqrt{area})$	Cumulative distribution function
μ	Location parameter of the GEV
δ	Scale parameter of the GEV
ξ	Shape parameter of the GEV
$P_{Occ}$	Probability of occurrence
$V_{0}$	Reference volume
EVIR	Extreme value inclusion rating
$V_{\alpha }$	Control volume
*T*	Return period
$F_{j}$	Cumulative probability
*j*	Index variable
*n*	Maximum index variable
$y_{j}$	Reduced variate
HV	Vickers hardness
$C_{1},C_{2}$	Coefficients of Murakami’s $\sqrt{area}$ concept
HIP	Hot isostatic pressing
DAS	Dendrite arm spacing
$l_{const}$	Length of constant specimen diameter
HCF	High cycle fatigue
$k_{1}$	Slope of S/N-curve in finite life region
$k_{2}$	Slope of S/N-curve in long-life region
$N_{T}$	Number of load cycles for transition point
$T_{S}$	Scatter band in the long-life region
$\kappa_{exp}$	Experimentally evaluated Weibull factor
$P_{S}$	Probability of survival
α	Return period of reference volume
$p_{ks}$	p-Value of Kolmogorov-Smirnov test
$\sqrt{area}P_{Occ=0.5}$	Defect size with a probability of occurrence of 50%
$\mu_{0}$	Reference defect distribution location parameter
Δ	Deviation of model to experiment
